# Emotional intelligence and language learning performance of EFL learners in China: chain mediating effects of willingness to communicate and foreign language learning boredom

**DOI:** 10.3389/fpsyg.2025.1702948

**Published:** 2026-01-13

**Authors:** Ruoting Wang, Qingshu Xu

**Affiliations:** 1 Foreign Studies College, Hunan Normal University, Changsha, China; 2 College of Education, University of Maryland, College Park, MD, United States

**Keywords:** boredom, emotional intelligence, language learning, mediation, motivation

## Abstract

**Introduction:**

Emotional intelligence (EI) has been widely recognized as a key affective factor in second language (L2) learning, yet its underlying mechanisms remain insufficiently explained. Drawing on Control–Value Theory (CVT), this study conceptualizes EI as a distal resource that enhances control–value appraisals, thereby fostering communicative engagement and mitigating negative achievement emotions, which jointly influence performance.

**Methods:**

A total of 1,158 Chinese undergraduates from seven universities completed validated measures of EI, willingness to communicate (WTC), foreign language learning boredom (FLLB), and submitted EFL test results. Structural equation modeling (SEM) was conducted using parcel-level indicators, and indirect effects were examined via bootstrap estimation (*B* = 5,000, BCa).

**Results:**

The hypothesized model demonstrated excellent fit and revealed that EI directly predicted language performance and indirectly influenced outcomes through both WTC and FLLB. Specifically, EI enhanced WTC, which reduced FLLB, and these factors jointly contributed to improved performance. Although the sequential (EI → WTC → FLLB → Performance) path was modest, it supported the theorized chain mediation mechanism consistent with CVT.

**Discussion:**

The findings provide conceptual evidence that EI promotes language learning success not only by increasing communicative willingness but also by buffering against boredom—a low-control, low-value emotion. This integrative perspective advances a process-oriented account of how affective and motivational systems interact in L2 learning. Practically, it suggests that classroom interventions combining emotional competence training, autonomy-supportive pedagogy, and value-enhancing communicative tasks may strengthen engagement while alleviating boredom.

## Introduction

In recent years, research on second language acquisition (SLA) has moved toward a broader consideration of affective variables, recognizing that emotions and interpersonal dispositions play a crucial role in learners’ success. One construct that has attracted consistent scholarly attention is emotional intelligence (EI), generally defined as the ability to perceive, understand, regulate, and utilize emotions in oneself and others (Dewaele et al., 2008; Li and Xu, 2019). EI encompasses key dimensions such as emotional attention, clarity, and repair (Li and Dewaele, 2020; Resnik and Dewaele, 2020). The three components, respectively, refer to, as is observed by Salovey et al. (1995, p. 127), “the degree of attention that the individuals devote to their feelings, the clarity of their experience of these feelings and their beliefs about terminating negative mood states or prolonging positive ones.” Prior research has demonstrated, under the theoretical guidance of Control-Value theory (Pekrun, 2006), EI’s impact on academic engagement (Aljasir, 2024), motivation (Chang and Tsai, 2022), and communicative success (Gao et al., 2025). Two other socio-affective factors—willingness to communicate (WTC) and foreign language learning boredom (FLLB), have been identified as particularly relevant to explaining how EI translates into language learning performance (Li and Dewaele, 2020; Zhang and Zhang, 2023). WTC refers to learners’ volitional readiness to initiate communication in the target language when opportunities arise (Asker, 1998; MacIntyre, 1994; MacIntyre et al., 2001; Macintyre et al., 1998; McCroskey and Baer, 1985). It is now regarded as one of the most powerful predictors of second language achievement, as learners with higher WTC are more likely to engage in authentic communicative practice and thereby accelerate their language development (Gao et al., 2025; Li Y., 2025). Research further indicates that WTC is not merely a trait but a dynamic construct influenced by situational factors, such as anxiety, attitudes, and the perceived quality of interaction (Feng et al., 2025; Leeming et al., 2024; Zhang et al., 2025).

Meanwhile, foreign language learning boredom is a negative, deactivating emotion characterized by dissatisfaction, inattention, and reduced vitality in classroom learning activities (Li, 2022; Pawlak et al., 2021; Wang and Xu, 2021). It often emerges when tasks are perceived as either over-challenging or under-challenging, and it is increasingly recognized as a significant barrier to learning. Recent structural equation modeling studies have shown that FLLB negatively correlates with engagement, motivation, and perceived achievement, making it a critical variable to examine alongside enjoyment and anxiety (Derakhshan et al., 2024; Li C. et al., 2023; Zheng and Feng, 2023). Particularly in exam-oriented environments such as China, FLLB can undermine learners’ motivation and communicative readiness, thus impeding language learning outcomes (Li K. et al., 2023; Liu and Dong, 2023).

While WTC and FLLB have typically been studied independently, they may form a sequential pathway through which EI influences EFL learners’ performance. Learners with stronger EI are more likely to manage anxiety and foster positive interpersonal interactions, which heightens their willingness to communicate (Li and Pan, 2025). Increased WTC, in turn, can reduce boredom by making language learning more meaningful, interactive, and rewarding. Conversely, low WTC may exacerbate boredom due to limited engagement opportunities. Thus, a chain mediation model—EI → WTC → FLLB → Performance—offers a promising framework for understanding the socio-emotional dynamics of language learning.

Despite their theoretical importance, limited empirical research has examined WTC and FLLB simultaneously as mediators between EI and EFL performance. Most existing studies have treated these constructs in isolation or explored their direct links to achievement. By investigating the sequential mediating roles of WTC and FLLB, this study seeks to fill this gap and to provide a more comprehensive picture of how EI translates into measurable language learning outcomes.

## Literature review

### Control-value theory

Anchoring this study is the Control–Value Theory of achievement emotions (CVT) (Pekrun, 2006), which explains how learners’ emotions emerge from their appraisals of control (perceived competence/causality) and value (importance, usefulness, or interest) in academic activities and outcomes. Within second/foreign language learning, CVT has been widely used to clarify links among emotional states (e.g., enjoyment, anxiety, boredom), their antecedents, and downstream outcomes such as willingness to communicate (WTC) (Ding and Wang, 2024; Wang et al., 2025).

In CVT, achievement emotions are shaped by proximal appraisals, specifically, perceived control, intrinsic value, and attainment value—that learners form toward a given task or outcome (Pekrun, 2006). These appraisals are themselves conditioned by distal antecedents, including relatively stable personal characteristics (e.g., trait/emotional intelligence) and features of the social–cultural environment (e.g., perceived teacher support) (Li C., 2025). CVT thus positions trait EI as a personality resource that filters how students construe control and value; teachers’ practices similarly scaffold appraisals and emotional experiences (Li et al., 2025). In turn, these emotions influence cognition and learning (e.g., via working memory, self-regulation, and metacognition), ultimately shaping language outcomes and communicative dispositions such as WTC (Li and Pan, 2025).

Guided by this framework, we conceptualize emotional intelligence (EI) as a distal antecedent that enhances favorable control–value appraisals in L2 learning; willingness to communicate (WTC) as a proximal, engagement-oriented outcome of these appraisals; and foreign language learning boredom (FLLB) as a negative achievement emotion that typically emerges when learners perceive low control and/or low value.

### Emotional intelligence and language learning performance

Emotional intelligence (EI) has been conceptualized as the ability to perceive, understand, regulate, and utilize emotions in oneself and others (Aljasir, 2024; Imamyartha et al., 2023; Li C. et al., 2023; Li et al., 2024; Piccerillo et al., 2025; Zhang, 2025). Scholars have distinguished between ability EI, which refers to emotion-related cognitive capacities, and trait EI, which reflects self-perceptions of emotional dispositions (Dewaele et al., 2008). A multidimensional approach also emphasizes emotional attention, clarity, and repair, which together enables learners to sustain adaptive behaviors and cope with challenges in academic and social contexts (Resnik et al., 2021).

A large body of research has documented the positive association between EI and academic achievement across disciplines (Chang and Tsai, 2022; MacCann et al., 2020; Yang and Duan, 2023). Learners with higher EI are more likely to manage stress (Zhang, 2025), maintain motivation (Chang and Tsai, 2022), and build constructive interpersonal relationships (Suprayogi and Andestia, 2023), which in turn foster better learning outcomes (Aljasir, 2024; Ding and Wang, 2024; Imamyartha et al., 2023; Piccerillo et al., 2025; Yang and Lin, 2024). In educational settings, EI has been linked to higher student engagement (Aljasir, 2024) and self-regulation (Taherkhani and Moradi, 2022). These findings suggest that the socio-emotional competencies captured by EI extend beyond personal well-being and directly support cognitive and academic development.

In the field of second language acquisition (SLA), EI has been increasingly recognized as a predictor of language learning success. Studies have shown that emotionally intelligent learners are more adept at regulating anxiety, coping with setbacks, and maintaining a positive orientation toward language learning (Dewaele et al., 2008; Li, 2020). Taherkhani and Moradi (2022) demonstrated that EI significantly predicted Persian foreign language learners’ reading comprehension, with learners high in EI displaying stronger language processing skills and reduced anxiety. These findings underscore EI’s role in facilitating both affective regulation and linguistic performance.

Research in the Chinese EFL context has provided additional evidence. Li C. et al. (2023) reported that higher EI enabled learners to mitigate negative emotions such as boredom and anxiety, leading to better engagement and perceived achievement. Zhang et al. (2025) further highlighted that teacher EI not only reduces learners’ stress but also indirectly enhances student performance through improved classroom atmosphere. Together, these studies suggest that EI contributes to language learning both directly—by equipping learners with emotional resources—and indirectly—by shaping the learning environment in favorable ways.

Given this evidence, it is reasonable to expect that Chinese EFL learners with higher emotional intelligence will demonstrate stronger language learning performance. Therefore, the first hypothesis is proposed:

*H1*: Emotional intelligence is positively linked to EFL learners’ language learning performance.

### Emotional intelligence and willingness to communicate

Willingness to communicate (WTC) in a second language is defined as a learner’s volitional readiness to initiate communication in the L2 when given the opportunity (Macintyre et al., 1998). Unlike early conceptualizations of WTC in the first language as a relatively stable trait (McCroskey and Baer, 1985), WTC in an L2 is now widely regarded as a dynamic, context-sensitive construct, shaped by situational, interpersonal, and emotional factors (Macintyre and Legatto, 2011; Wang et al., 2025). Research has consistently shown that WTC is one of the most proximal predictors of communicative behavior and thus a key determinant of L2 development. Learners with higher WTC are more likely to engage in authentic practice, which in turn enhances proficiency and confidence.

Emotional intelligence (EI) plays an important role in fostering WTC. Learners with higher EI are better able to manage communication anxiety (Birjandi and Tabataba'ian, 2012), regulate negative emotions (Babanoglu, 2025), and perceive social cues (Piccerillo et al., 2025), all of which support greater readiness to speak. As Dewaele and Pavelescu (2021) observed in multiple case studies, emotions such as enjoyment, anxiety, and boredom closely interact with WTC, often amplifying or suppressing learners’ willingness to participate. In this sense, EI functions as a resource that allows learners to navigate these affective experiences more effectively.

Recent empirical studies provide evidence of the EI–WTC link. Taherkhani and Moradi (2022), using structural equation modeling with Persian foreign language learners, found that EI significantly predicted learners’ WTC, which in turn strongly predicted reading comprehension performance. Similarly, other research has shown that higher EI enhances learners’ interpersonal skills, empathy, and confidence, all of which contribute to greater communicative engagement in the L2 classroom (Oz, 2014). Interviews with learners further confirm that those capable of controlling emotions and understanding others’ feelings report higher tendencies to engage in communication, particularly in supportive classroom contexts (De Neve et al., 2023).

In EFL context, where learners might experience high levels of foreign language classroom anxiety, EI can be especially important in enabling learners to cope with stress and overcome reluctance to speak (Jin et al., 2024). Zhang et al. (2025) reported that teacher EI fosters a positive emotional climate that indirectly boosts students’ communicative engagement. Li et al. (2021) also found that emotionally intelligent learners are better at reducing negative emotions, thereby sustaining their willingness to communicate in the classroom.

Taken together, these findings suggest that EI enhances learners’ willingness to communicate by equipping them with emotional regulation skills and positive interpersonal dispositions. Learners with higher EI are thus more likely to participate actively in communicative tasks and engage meaningfully with peers and teachers in the EFL classroom.

*H2*: Emotional intelligence is positively linked to willingness to communicate among EFL learners.

### Willingness to communicate and foreign language learning boredom

Foreign language learning boredom (FLLB) is defined as a negative, low-arousal emotion characterized by dissatisfaction, inattention, and reduced motivation (Kruk and Zawodniak, 2018). It often arises when learning activities are perceived as either over-challenging or under-challenging. Recent research has demonstrated that FLLB is strongly intertwined with other classroom emotions: it is negatively associated with enjoyment and positively associated with anxiety, suggesting that emotions interact dynamically rather than operating in isolation (Botes et al., 2021; Botes et al., 2022). Studies indicate that communicative engagement and boredom are inversely related. For instance, structural equation modeling (SEM) with foreign language learners revealed that teacher unpredictability—introducing variety into classroom activities—was positively associated with enjoyment but negatively associated with boredom (Dewaele et al., 2023). This finding shows that classroom conditions which stimulate more participation and interaction reduce boredom, pointing indirectly to a negative WTC–FLLB link (Bai, 2023).

While one might argue that FLLB could also reduce students’ WTC, we conceptualize the causal direction from WTC to FLLB based on both theoretical and empirical grounds. According to Control–Value Theory (Pekrun, 2006), boredom arises primarily from low appraisals of control and value. WTC, as a motivational–behavioral disposition, reflects learners’ readiness to engage in communication when they perceive high control and high value. Thus, higher WTC should protect against boredom by fostering greater classroom involvement. Moreover, from a dynamic systems perspective (Gregersen and MacIntyre, 2014), motivational intentions such as WTC tend to precede and shape learners’ emotional experiences like FLLB *in situ*. FLLB does not have fixed effects, and it interacts with motivational behaviors and that interaction changes over time (de Bot, 2016). Further evidence comes from dynamic and moment-to-moment research. Kruk et al. (2022) tracked fluctuations of WTC, anxiety, and boredom over time and found that these variables co-varied during lessons: when WTC and engagement increased, boredom tended to decrease.

In the Chinese EFL context, where exam-oriented instruction often limits authentic communicative opportunities, boredom has been reported as a widespread challenge (Li and Wei, 2022). Under such conditions, learners with higher WTC are more likely to engage in classroom activities, thereby counteracting boredom and maintaining active involvement.

*H3*: Willingness to communicate is negatively linked to foreign language learning boredom among EFL learners.

### Foreign language learning boredom and language learning performance

Foreign language learning boredom (FLLB) has recently been recognized as a salient negative classroom emotion in applied linguistics. It is typically defined as a deactivating, low-arousal emotional state characterized by dissatisfaction, lack of motivation, and reduced vitality in learning activities (Kruk and Zawodniak, 2018). According to control–value theory (Pekrun, 2006), boredom arises when learners perceive tasks as either over-challenging or under-challenging, which undermines both concentration and engagement. In the foreign language classroom, FLLB reduces learners’ attention and willingness to invest effort, thereby limiting opportunities for meaningful practice.

A growing body of studies has demonstrated the detrimental impact of FLLB on language learning outcomes. Structural equation modeling studies have found that FLLB correlates negatively with learners’ motivation, engagement, and perceived achievement (Dewaele et al., 2023; Li, 2021). For instance, Li and Wei (2022) showed that Chinese EFL learners who reported higher levels of FLLB displayed lower levels of both perceived competence and actual achievement over time. Similarly, Li and Han (2022) reported that FLLB predicted lower academic outcomes, particularly when coupled with high anxiety. A recent meta-analysis also confirms that, while enjoyment fosters achievement and anxiety undermines it, boredom consistently emerges as an additional negative predictor of both actual and self-perceived performance (Teimouri et al., 2019). Importantly, although the negative effect of FLLB on achievement sometimes weakens over time compared to the stronger and more enduring impact of anxiety (Li and Wei, 2022), its immediate effect on learners’ performance and engagement remains robust. Literature consistently demonstrates that FLLB undermines learners’ focus, motivation, and active participation, thereby impairing language learning outcomes (Li and Wei, 2022). When learners experience boredom in language classes, their capacity to engage in sustained learning and to perform successfully in assessments is diminished.

*H4*: Foreign language learning boredom is negatively linked to language learning performance among EFL learners.

### Willingness to communicate and language learning performance

A substantial body of research has confirmed the link between WTC and L2 achievement. Learners with higher WTC are more likely to engage in authentic interaction, which enhances opportunities for practice and fosters skill development across speaking, listening, reading, and writing (Dörnyei, 2005). For example, Taherkhani and Moradi (2022) found that WTC was the strongest predictor of Persian foreign language learners’ reading comprehension in their structural equation model, even surpassing emotional intelligence and self-regulation. These studies indicate that WTC is a key facilitator of second language performance. By enabling learners to seize communicative opportunities, WTC expands practice, reduces passivity, and fosters achievement across multiple skills. Learners with higher WTC are thus expected to demonstrate better EFL performance than their less communicative peers.

*H5*: Willingness to communicate is positively linked to language learning performance among EFL learners.

### Emotional intelligence and foreign language learning boredom

Although the link between emotional intelligence and boredom has received comparatively less attention than its connection to engagement or motivation, theory and emerging evidence suggest that EI should negatively predict foreign language learning boredom (FLLB). According to Control–Value Theory (Pekrun, 2006), boredom arises when learners experience low control over learning activities or assign low value to them. Learners with higher EI are better able to regulate negative emotions, reinterpret challenges, and sustain a sense of personal relevance, thereby maintaining both perceived control and task value (Dewaele et al., 2023). In communicative classrooms, such learners can manage frustration stemming from repetitive or difficult exercises, shifting attention from negative emotions toward goal-directed engagement. Empirical studies in general education contexts have also shown that emotionally intelligent students report less boredom and higher enjoyment because they possess stronger coping and reappraisal strategies (Derakhshan et al., 2024). Therefore, it is plausible that EI functions as a protective emotional resource, helping EFL learners to remain attentive, motivated, and involved even under monotonous or demanding instructional conditions. Accordingly, the present study hypothesizes that EI is negatively associated with FLLB.

*H6*: Emotional Intelligence is negatively linked to foreign language learning boredom (FLLB).

### The chain mediating role of willingness to communicate and foreign language learning boredom

While emotional intelligence (EI), willingness to communicate (WTC), and foreign language learning boredom (FLLB) have been studied as independent predictors of language learning, recent advances in applied linguistics and educational psychology emphasize the importance of examining how these constructs interact dynamically. EI enables learners to recognize, regulate, and utilize emotions effectively. WTC reflects learners’ volitional readiness to engage in communication, and FLLB represents a deactivating negative emotion marked by disengagement and low motivation. Taken together, these constructs suggest a potential sequential pathway: emotionally intelligent learners are more likely to communicate, which in turn reduces their experience of boredom, ultimately enhancing their performance.

Prior studies have consistently demonstrated the positive effect of EI on WTC. Learners with higher EI manage anxiety better, empathize with others, and regulate emotions in communicative settings, all of which promote greater readiness to interact (Taherkhani and Moradi, 2022). At the same time, classroom-based research has shown that increased participation and communicative engagement tend to reduce boredom, as interaction provides novelty, variety, and meaningfulness in language use (Dewaele et al., 2023). Conversely, when learners avoid communication opportunities, they are more likely to perceive tasks as monotonous or irrelevant, which intensifies FLLB (Li, 2021).

Importantly, the relationship between boredom and achievement has also been empirically supported. FLLB is negatively associated with motivation, engagement, and academic outcomes (Teimouri et al., 2019). In contrast, WTC has been identified as one of the strongest predictors of language performance across multiple skills. When considered together, these findings imply that WTC and FLLB may serve as sequential mediators: EI fosters communicative willingness, which decreases boredom, and the reduction in boredom then contributes to stronger learning outcomes.

*H7*: Willingness to communicate (WTC) and foreign language learning boredom (FLLB) sequentially mediate the relationship between emotional intelligence and language learning performance among EFL learners.

**Figure 1:**
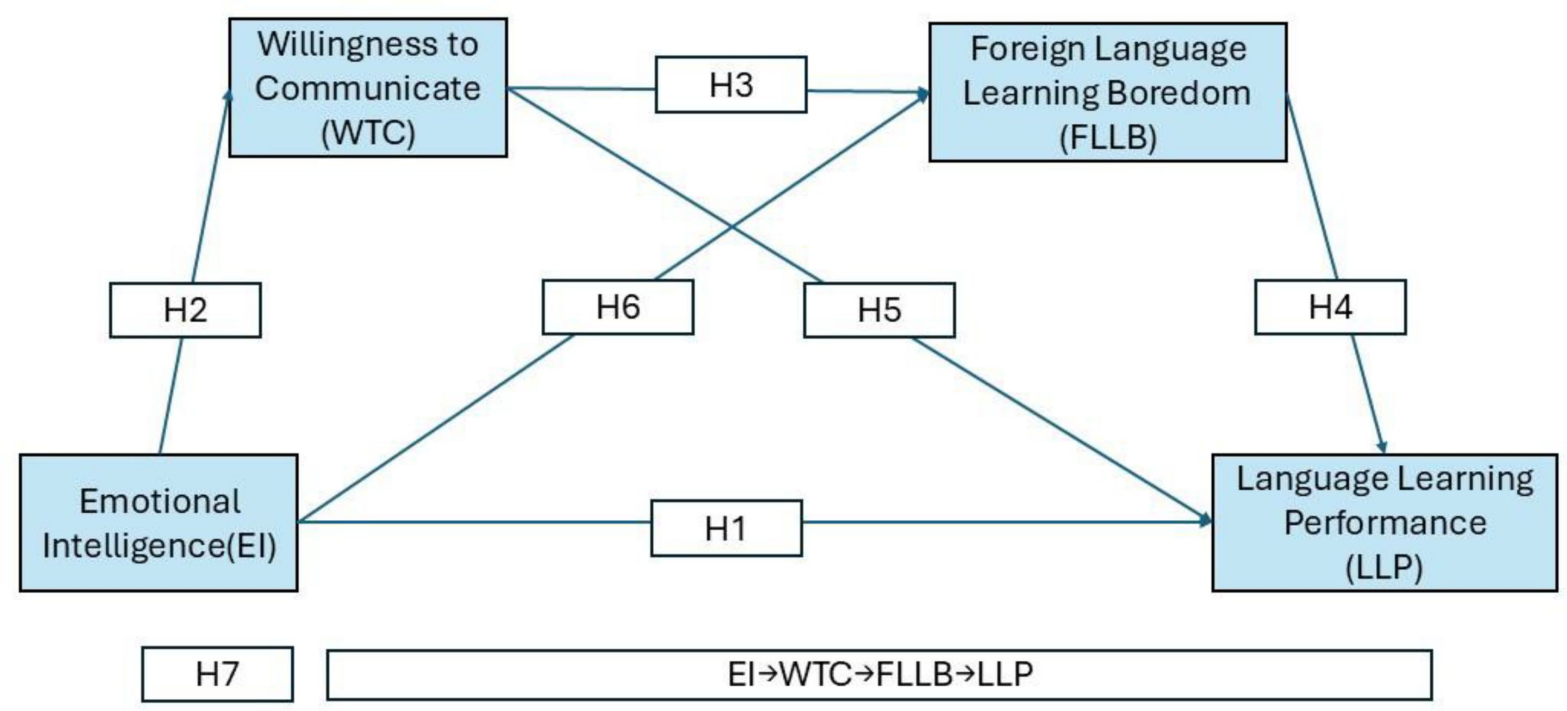
Proposed chain mediation model of the relationship between emotional intelligence, willingness to communicate, foreign language learning boredom, and EFL learners’ language learning performance.

The proposed model illustrates the pathway through which emotional intelligence influences EFL learners’ language learning performance, with willingness to communicate and foreign language learning boredom serving as sequential mediators, as depicted in Figure 1. Specifically, this research addresses three questions: (1) Does EI significantly predict EFL learners’ language learning performance? (2) Do WTC and FLLB mediate this relationship independently? (3) Does a chain mediation model better explain the pathways from EI to performance? The findings will extend theoretical insights into affective-motivational processes in SLA and provide practical implications for reducing boredom and fostering communication in EFL classrooms.

## Methods

### Participants

The participants in this study were undergraduate students from seven public tertiary institutions across China, including four universities located in East China and three in Central China, all offering standard four-year undergraduate programs. This diverse institutional coverage provides a broad representation of EFL learners across different regions of the country. A total of 1,372 students completed the online questionnaire distributed via a self-developed secure survey platform. After excluding 214 invalid responses due to insufficient response time or missing data, 1,158 valid questionnaires were retained for analysis. Among the valid respondents, 606 were male (52.3%) and 552 were female (47.7%).

The sample included students from all 4 years of undergraduate study: freshmen (*n* = 403), sophomores (*n* = 312), juniors (*n* = 385), and seniors (*n* = 58). The relatively small number of senior participants can be explained by their heavier engagement in employment preparation and thesis writing during the period of data collection. The average age of the participants was 20.2 years (SD = 0.7), which is typical of undergraduate cohorts in China.

All participants were non-English majors enrolled in compulsory English as a Foreign Language (EFL) course, ensuring a relatively comparable learning background. They had studied English for an average of 8–10 years prior to entering university. Participation was voluntary, and students were informed that their responses would remain anonymous and would not influence their academic standing. Informed consent was obtained electronically before they began the questionnaire.

### Setting

This study was conducted in the context of undergraduate English education in China. At the participating universities, English is a compulsory subject for all non-English majors during their first 2 years of study, with many students continuing to take English-related courses in their third and fourth years. The courses are typically delivered in large lecture-style classes, supplemented by smaller discussion sessions or online platforms, and are strongly oriented toward standardized assessments such as the College English Test Band 4 (CET-4).

Instruction in these courses generally emphasizes reading, writing, listening, and test-taking skills, while opportunities for authentic communicative practice are relatively limited. As a result, learners often experience anxiety and boredom in classroom settings, and their willingness to communicate varies considerably depending on task type and classroom climate. This environment makes it particularly suitable for investigating the relationships among emotional intelligence (EI), willingness to communicate (WTC), foreign language learning boredom (FLLB), and language learning performance.

### Instruments

Four instruments were employed to measure the constructs of interest in this study: emotional intelligence (EI), willingness to communicate (WTC), foreign language learning boredom (FLLB), and language learning performance. All scales were administered in Chinese to ensure clarity and accuracy, and a standard back-translation procedure was used to confirm linguistic equivalence. Prior to the formal survey, a pilot test with a small sample of students (n = 57) was conducted to ensure the reliability and comprehensibility of the instruments.

1 Emotional intelligence

Emotional intelligence was measured using the Wong and Law Emotional Intelligence Scale (WLEIS) (Wong and Law, 2002). The scale consists of 16 items covering four dimensions: (a) self-emotion appraisal (“I have good understanding of my own emotions.”), (b) others’ emotion appraisal (“I am a good observer of others’ emotions.”), (c) use of emotion (“I always tell myself I am a competent person.”), and (d) regulation of emotion (“I am quite capable of controlling my own emotions.”). Items were rated on a five-point Likert scale ranging from 1 (strongly disagree) to 5 (strongly agree). Higher scores indicate greater perceived emotional intelligence. The WLEIS has been widely validated in different cultural contexts, including China, and has demonstrated strong reliability (Cronbach’s *α* typically > 0.80). In the present study, Cronbach’s α for the overall scale was approximately 0.89.

2 Willingness to communicate (WTC)

Willingness to communicate in English was measured using an adapted version of Weaver’s (2005) WTC scale for speaking. The adaptation was carried out by Peng and Woodrow (2010), who selected 10 items from the original 15 items based on item analysis and contextual relevance. The 10 retained items were rated on a six-point Likert scale ranging from 1 (strongly disagree) to 6 (strongly agree) and represented two dimensions: (1) willingness to communicate in English during meaning-focused activities (6 items, e.g., “I am willing to give a short self-introduction without notes in English to the class.”), and (2) willingness to communicate in English during form-focused activities (4 items, e.g., “I am willing to ask my peer sitting next to me in English the meaning of an English word.”). Higher scores indicated greater willingness to communicate in the target language. The scale has demonstrated good psychometric properties in prior research. Peng and Woodrow (2010) reported satisfactory internal consistency, with Cronbach’s α = 0.88. In the present study, the WTC scale again showed strong reliability, with Cronbach’s α of approximately 0.84 for the overall scale and acceptable coefficients across the two subdimensions (0.86 and 0.84 respectively).

3 Foreign language learning boredom (FLLB)

The level of boredom experienced by learners in their English classes was assessed using the Foreign Language Learning Boredom Scale (FLLBS) developed by Li et al. (2021). The FLLBS was originally validated in a large-scale study among more than 3,000 Chinese university students and has since been widely used in EFL contexts. The scale consists of 32 items (like: The English class bores me.) grouped into seven factors: (1) Foreign Language Classroom Boredom (8 items, e.g., “The English class bores me.”), (2) Under-Challenging Task Boredom (5 items, e.g., “I believe an analysis of long text in English is really dreary.”), (3) PowerPoint Presentation Boredom (3 items, e.g., “Reading from script in the PPT slides bores me.”), (4) Homework Boredom (4 items, e.g., “Doing English homework is a dull activity.”), (5) Teacher-Dislike Boredom (4 items, e.g., “The English teacher is an uninteresting, so the English class is dull.”), (6) General Learning Trait Boredom (5 items, e.g., “I’m always bored when I study.”), and (7) Over-Challenging or Meaningless Task Boredom (3 items, e.g., “If I cannot understand classmates’ presentations, I become really bored.”). Participants rated all items on a five-point Likert scale ranging from 1 (strongly disagree) to 5 (strongly agree), with higher scores reflecting greater levels of boredom. The FLLBS has demonstrated excellent psychometric properties in the present study with global Cronbach’s *α* of 0.94 and strong reliability across the seven subscales (ranging from.73 to 0.89).

4 Language learning performance

Learners’ performance was operationalized as their final course grade in compulsory university English courses at their home institutions. Grades were reported on a 100-point scale, with higher values indicating better achievement. The recorded final grade was the official composite calculated by each university (e.g., based on quizzes, assignments, midterm/final exams per the course syllabus). Scores were submitted by the students. The universities where the samples are collected are of the same level in terms of college entrance examination scores and the test formats and difficulty are all aligned with the CSE intermediate levels.

### Procedure

Data collection was conducted during the spring semester of 2025. After obtaining approval from the institutional ethics committee, the research team contacted seven participating universities across China and invited undergraduate students to voluntarily take part in the study. The survey was distributed via a secure online questionnaire platform, which allowed for large-scale participation across multiple institutions. The students completed the online survey independently outside class time.

Before beginning the survey, participants read an informed consent form outlining the purpose of the study, confidentiality of responses, and their right to withdraw at any time without penalty. Only those who gave electronic consent were able to proceed. The questionnaire included measures of emotional intelligence (EI), willingness to communicate (WTC), and foreign language learning boredom (FLLB), followed by demographic questions. On average, it took participants about 20 min to complete the survey.

### Data analysis

All analyses were conducted using R (version 4.4.3). Data screening was performed to exclude incomplete responses, patterned answers, and implausibly short completion times, resulting in 1,158 valid cases. Descriptive statistics (means, standard deviations, skewness, and kurtosis) were obtained using the psych package to examine the distributional properties of the data.

The reliability of each scale was assessed through Cronbach’s α and McDonald’s *ω*, computed using the psych package. Construct validity was evaluated through confirmatory factor analysis (CFA) using the lavaan package. Convergent validity was examined via standardized factor loadings and average variance extracted (AVE), while discriminant validity was assessed using the Fornell–Larcker criterion. Model fit was evaluated with multiple indices, including χ^2^/df, Comparative Fit Index (CFI), Tucker–Lewis Index (TLI), Root Mean Square Error of Approximation (RMSEA), and Standardized Root Mean Square Residual (SRMR).

Because several constructs (e.g., EI, WTC, and FLLB) consisted of multiple items and dimensions, item parceling was employed to reduce model complexity and enhance measurement reliability. Following the recommendations of Little et al. (2002), items that demonstrated unidimensionality in exploratory and confirmatory factor analyses were aggregated into parcels, which then served as manifest indicators of the latent variables in the SEM analysis. This approach has the advantage of improving the stability of parameter estimates, reducing random measurement error, and producing more parsimonious models, while retaining the substantive meaning of the constructs.

**Figure 2:**
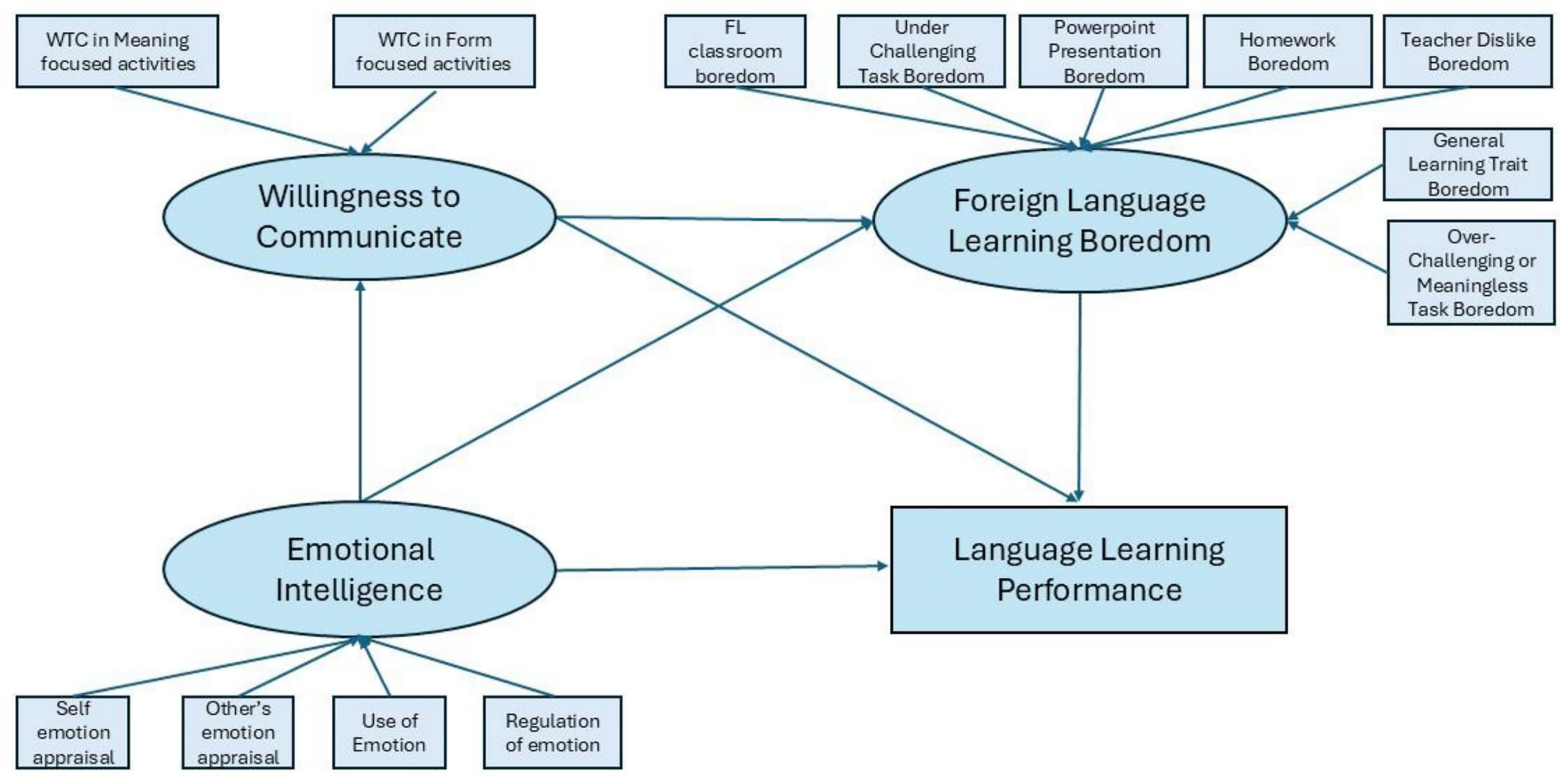
Measurement and structural model of the study.

After validating the measurement model, structural equation modeling (SEM) was conducted using lavaan to test the hypothesized paths among emotional intelligence (EI), willingness to communicate (WTC), foreign language learning boredom (FLLB), and language learning performance. Both direct and indirect effects were estimated as is shown in Figure 2.

The mediating roles of WTC and FLLB were further examined using bootstrapping procedures with 5,000 resamples, as implemented in semTools. Mediation effects were deemed significant when the 95% bias-corrected confidence intervals did not include zero. The chain mediation pathway (EI → WTC → FLLB → Performance) was tested alongside the individual mediating effects.

## Results

### Preliminary analysis

Descriptive statistics for the latent constructs and their subscales are presented in Tables 1, 2. At the construct level (Table 1), emotional intelligence (EI) had a mean score of 2.99 (SD = 0.98) on a five-point scale, indicating that participants generally perceived themselves as moderately emotionally intelligent. Willingness to communicate (WTC) averaged 3.51 (SD = 1.27) on a six-point scale, suggesting a moderate to high level of communicative engagement. Foreign language learning boredom (FLLB) showed a mean of 3.00 (SD = 0.97) on a five-point scale, reflecting a moderate degree of boredom. The performance variable, measured on a 100-point scale, had a mean score of 49.27 (SD = 15.09), showing substantial variability in language learning outcomes. For all constructs, skewness and kurtosis values were close to zero, ranging between −0.97 and 0.05, indicating no serious deviation from normality.

**Table 1:** Descriptive statistics for latent constructs.

Construct	*N*	*M*	*SD*	Min	Max	Skew	Kurt
EI	1,158	2.99	0.98	1	5	0.05	−0.97
WTC	1,158	3.51	1.27	1	6	−0.02	−0.96
FLLB	1,158	3.00	0.97	1	5	0.02	−0.94
Performance	1,158	49.27	15.09	0	100	0.00	−0.14

EI = Emotional Intelligence; WTC = Willingness to Communicate; FLLB = Foreign Language Learning Boredom; Performance = Language Learning Performance. Scores represent the mean of their respective subscales. N = sample size; M = mean; SD = standard deviation.

**Table 2:** Descriptive statistics for subscales.

Subscale	*N*	*M*	*SD*	Min	Max	Skew	Kurt
SEA	1,158	2.99	1.12	1	5	0.01	−1.02
OEA	1,158	2.99	1.14	1	5	0.02	−1.09
UOE	1,158	3.00	1.15	1	5	0.00	−1.09
ROE	1,158	2.99	1.10	1	5	0.01	−1.00
WTC1	1,158	3.50	1.32	1	6	0.01	−0.94
WTC2	1,158	3.51	1.41	1	6	−0.03	−1.09
FLCB	1,158	2.99	1.04	1	5	0.01	−0.99
UCTB	1,158	3.00	1.12	1	5	0.02	−1.04
PPB	1,158	3.00	1.14	1	5	0.05	−1.05
HWB	1,158	2.99	1.15	1	5	0.02	−1.09
TDB	1,158	3.00	1.15	1	5	0.02	−1.03
GLTB	1,158	3.00	1.07	1	5	0.02	−0.96
OCMTB	1,158	3.01	1.22	1	5	0.00	−1.14

EI = Emotional Intelligence; SEA = Self-Emotion Appraisal; OEA = Others’ Emotion Appraisal; UOE = Use of Emotion; ROE = Regulation of Emotion. WTC = Willingness to Communicate; WTC1 = Meaning-focused activities; WTC2 = Form-focused activities. FLLB = Foreign Language Learning Boredom; FLCB = Foreign Language Classroom Boredom; UCTB = Under-Challenging Task Boredom; PPB = PowerPoint Presentation Boredom; HWB = Homework Boredom; TDB = Teacher-Dislike Boredom; GLTB = General Learning Trait Boredom; OCMTB = Over-Challenging or Meaningless Task Boredom. N = sample size; M = mean; SD = standard deviation.

At the subscale level (Table 2), the four EI subscales (SEA, OEA, UOE, ROE) all had mean scores around 2.99–3.00 (SDs ≈ 1.10–1.15), reflecting moderate levels across the different emotional intelligence dimensions. The two WTC subscales (WTC1, WTC2) had slightly higher means of 3.50 and 3.51, with somewhat larger variability (SD = 1.32 and 1.41, respectively), suggesting that learners differed in their willingness to communicate in meaning-focused and form-focused classroom activities. The seven FLLB subscales yielded means close to 3.00 (range = 2.99–3.01, SDs ≈ 1.04–1.22), indicating consistent levels of boredom across different sources such as classroom activities, tasks, and teacher-related factors. Across all subscales, skewness and kurtosis values were again acceptable (−1.14 – 0.05) supporting the assumption of fitness for SEM in the data (West et al., 1995).

Correlations among the latent constructs are presented in Table 3. Emotional intelligence (EI) was positively correlated with willingness to communicate (WTC; *r* = 0.29, *p* < 0.001) and negatively correlated with foreign language learning boredom (FLLB; *r* = −0.27, *p* < 0.001). WTC was negatively correlated with FLLB (*r* = −0.32, *p* < 0.001). All three latent constructs were significantly associated with language learning performance: EI (*r* = 0.29, *p* < 0.001), WTC (*r* = 0.37, *p* < 0.001), and FLLB (*r* = −0.40, *p* < 0.001). These findings provide initial support for the hypothesized model, suggesting that learners with higher EI are more willing to communicate, less likely to experience boredom, and achieve better language performance.

**Table 3:** Correlations among latent constructs.

Construct	EI	WTC	FLLB	Performance
EI	1.00			
WTC	0.29***	1.00		
FLLB	−0.27***	−0.32***	1.00	
Performance	0.29***	0.37***	−0.40***	1.00

EI = Emotional Intelligence; WTC = Willingness to Communicate; FLLB = Foreign Language Learning Boredom; Performance = Language Learning Performance. Stars denote two-tailed significance: *p < 0.05, **p < 0.01, ***p < 0.001.

Table 4 displays the correlations among the subscales. The four EI subscales (SEA, OEA, UOE, ROE) were strongly and positively interrelated (*r*s = 0.66–0.69, *p*s < 0.001), confirming the coherence of the EI construct. The two WTC subscales (WTC1 and WTC2) were also highly correlated (*r* = 0.73, *p* < 0.001), indicating coherence across meaning-focused and form-focused WTC activities. The seven FLLB subscales were all significantly correlated with one another (*r*s = 0.65–0.73, *p*s < 0.001), suggesting that different sources of boredom were interrelated.

**Table 4:** Correlations among subscales.

Subscale	SEA	OEA	UOE	ROE	WTC1	WTC2	FLCB	UCTB	PPB	HWB	TDB	GLTB	OCMTB
SEA	1.00												
OEA	0.68***	1.00											
UOE	0.69***	0.67***	1.00										
ROE	0.66***	0.67***	0.67***	1.00									
WTC1	0.24***	0.24***	0.23***	0.26***	1.00								
WTC2	0.23***	0.22***	0.22***	0.23***	0.73***	1.00							
FLCB	−0.23***	−0.24***	−0.21***	−0.22***	−0.27***	−0.26***	1.00						
UCTB	−0.17***	−0.19***	−0.20***	−0.21***	−0.25***	−0.24***	0.71***	1.00					
PPB	−0.17***	−0.18***	−0.18***	−0.18***	−0.24***	−0.26***	0.70***	0.67***	1.00				
HWB	−0.23***	−0.23***	−0.22***	−0.22***	−0.26***	−0.26***	0.73***	0.69***	0.68***	1.00			
TDB	−0.18***	−0.17***	−0.18***	−0.19***	−0.24***	−0.25***	0.72***	0.69***	0.67***	0.72***	1.00		
GLTB	−0.22***	−0.21***	−0.21***	−0.22***	−0.27***	−0.25***	0.69***	0.72***	0.66***	0.71***	0.69***	1.00	
OCMTB	−0.22***	−0.21***	−0.23***	−0.23***	−0.25***	−0.25***	0.70***	0.71***	0.65***	0.69***	0.72***	0.70***	1.00

Pearson correlations among subscales. SEA = Self-Emotion Appraisal; OEA = Others’ Emotion Appraisal; UOE = Use of Emotion; ROE = Regulation of Emotion; WTC1 = Meaning-focused activities; WTC2 = Form-focused activities; FLCB = Foreign Language Classroom Boredom; UCTB = Under-Challenging Task Boredom; PPB = PowerPoint Presentation Boredom; HWB = Homework Boredom; TDB = Teacher-Dislike Boredom; GLTB = General Learning Trait Boredom; OCMTB = Over-Challenging or Meaningless Task Boredom. Stars denote two-tailed significance: *p < 0.05, **p < 0.01, ***p < 0.001.

Cross-constructs correlations revealed theoretically consistent patterns. EI subscales were positively correlated with WTC subscales (*r*s ≈ 0.22–0.26, *p*s < 0.001) and negatively correlated with FLLB subscales (*r*s ≈ −0.17 to −0.23, *p*s < 0.001). WTC subscales were negatively associated with all FLLB subscales (*r*s ≈ −0.24 to −0.27, *p*s < 0.001). These correlations reinforce the theoretical expectations that EI facilitates greater willingness to communicate and mitigates boredom, while WTC further reduces boredom, all of which are linked to enhanced performance.

### Validity and reliability

Convergent validity and reliability indices for all latent constructs and subscales are presented in Table 5. For Average Variance Extracted (AVE), the common minimum threshold is 0.5 or greater, indicating sufficient convergent validity and for Composite Reliability (CR), the accepted threshold is 0.7 or greater, indicating adequate reliability for a construct (Cheung et al., 2024). At the latent construct level, results demonstrated strong psychometric properties. Emotional intelligence (EI) showed an AVE of 0.673 and CR of 0.892, with *ω* and *α* both equal to 0.892, indicating excellent convergent validity and reliability. Willingness to communicate (WTC) also showed high values (AVE = 0.727, CR = 0.842, ω = 0.841, α = 0.840), despite having only two subscales as indicators. Foreign language learning boredom (FLLB) exhibited the strongest validity indices, with AVE = 0.696, CR = 0.941, and ω and α both at 0.941. All three latent variables exceeded the recommended thresholds (AVE > 0.50, CR > 0.70, α > 0.70), confirming good convergent validity and reliability.

**Table 5:** Validity and reliability for latent constructs and subscales.

Level	Construct	*k* (indicators)	AVE	CR	*ω* (Omega)	*α* (Alpha)
Latent	EI	4	0.673	0.892	0.892	0.892
Latent	FLLB	7	0.696	0.941	0.941	0.941
Latent	WTC	2	0.727	0.842	0.841	0.840
EI	OEA	4	0.537	0.822	0.794	0.793
EI	ROE	4	0.512	0.807	0.775	0.775
EI	SEA	4	0.524	0.815	0.782	0.782
EI	UOE	4	0.557	0.834	0.801	0.801
WTC	WTC1	6	0.557	0.883	0.864	0.864
WTC	WTC2	4	0.604	0.859	0.836	0.836
FLLB	FLCB	8	0.545	0.906	0.886	0.886
FLLB	GLTB	5	0.501	0.834	0.805	0.805
FLLB	HWB	4	0.589	0.851	0.822	0.822
FLLB	OCMTB	3	0.682	0.865	0.835	0.835
FLLB	PPB	3	0.532	0.773	0.735	0.734
FLLB	TDB	4	0.601	0.858	0.828	0.828
FLLB	UCTB	5	0.597	0.881	0.857	0.857

AVE = Average Variance Extracted; CR = Composite Reliability; ω = McDonald’s Omega; α = Cronbach’s alpha; k = number of indicators (items for subscales; subscales for latent constructs). In the Level column, “Latent” rows report indices for the higher-order constructs (EI, WTC, FLLB) estimated from their subscale indicators; for subscale rows, Level indicates the parent latent variable (EI, WTC, or FLLB) that the subscale belongs to.

At the subscale level, the EI subscales (SEA, OEA, UOE, ROE) reported AVE values between 0.512 and 0.557, CR between 0.807 and 0.834, and reliability indices (ω, α) around 0.78–0.80, all within acceptable ranges. The two WTC subscales also demonstrated good measurement properties: WTC1 (AVE = 0.557, CR = 0.883, *ω* = 0.864, *α* = 0.864) and WTC2 (AVE = 0.604, CR = 0.859, *ω* = 0.836, *α* = 0.836). For the FLLB subscales, AVE values ranged from 0.501 (GLTB) to 0.682 (OCMTB), with CR between 0.773 (PPB) and 0.906 (FLCB). Reliability indices were consistently high, with ω and α ranging from 0.734 to 0.886.

These results indicate that the measurement instruments for EI, WTC, and FLLB demonstrated strong reliability and satisfactory convergent validity at both the latent and subscale levels. The findings support the adequacy of the measurement model and provide a solid basis for subsequent structural model testing.

### Measurement model and structural model results

Before item parceling, we used CFA methods to check the unidimensionality of the subscale items and the loadings for each subscale item are all above 0.50 and indexes like TFI/CLI, RMSEA, SRMR all meet the criteria. Then, the measurement model using subscale parcels was evaluated through confirmatory factor analysis (CFA). As shown in Table 6, all indicators loaded strongly and significantly on their respective latent constructs. For emotional intelligence (EI), factor loadings ranged from 0.814 (ROE) to 0.828 (SEA), all highly significant (*p*s < 0.001). For foreign language learning boredom (FLLB), loadings ranged from 0.812 (PPB) to 0.871 (FLCB), again with all *p*s < 0.001. For willingness to communicate (WTC), both subscales loaded substantially on the construct (WTC1 = 0.894, WTC2 = 0.846, both *p*s < 0.001). These results demonstrate that the parcels served as reliable indicators of their intended latent variables.

**Table 6:** Measurement model (subscale parcels): standardized factor loadings.

Factor	Indicator	Loading	SE	*z*	*p*
EI	SEA	0.828	0.012	70.151	<0.001
EI	OEA	0.826	0.012	69.613	<0.001
EI	UOE	0.820	0.012	67.862	<0.001
EI	ROE	0.814	0.012	66.069	<0.001
FLLB	FLCB	0.871	0.008	107.693	<0.001
FLLB	HWB	0.858	0.009	98.033	<0.001
FLLB	UCTB	0.857	0.009	97.678	<0.001
FLLB	GLTB	0.850	0.009	93.008	<0.001
FLLB	TDB	0.848	0.009	92.201	<0.001
FLLB	OCMTB	0.847	0.009	91.287	<0.001
FLLB	PPB	0.812	0.011	74.293	<0.001
WTC	WTC1	0.894	0.024	36.544	<0.001
WTC	WTC2	0.846	0.024	35.297	<0.001

Standardized factor loadings (λ), standard errors (SE), z-values, and two-tailed *p*-values are reported. SEA = Self-Emotion Appraisal; OEA = Others’ Emotion Appraisal; UOE = Use of Emotion; ROE = Regulation of Emotion; WTC1 = Meaning-focused activities; WTC2 = Form-focused activities; FLCB = Foreign Language Classroom Boredom; UCTB = Under-Challenging Task Boredom; PPB = PowerPoint Presentation Boredom; HWB = Homework Boredom; TDB = Teacher-Dislike Boredom; GLTB = General Learning Trait Boredom; OCMTB = Over-Challenging or Meaningless Task Boredom; EI = Emotional Intelligence; WTC = Willingness to Communicate; FLLB = Foreign Language Learning Boredom.

The overall measurement model also exhibited an excellent fit to the data: χ^2^(62) = 97.48, *p* = 0.003, CFI = 0.997, TLI = 0.996, RMSEA = 0.022 (90% CI [0.013, 0.030]), and SRMR = 0.013. All indices met or exceeded conventional thresholds —CFI ≥ 0.95, TLI ≥ 0.95, RMSEA ≤ 0.06 with the 90% CI upper bound ≤ 0.08, and SRMR ≤ 0.08 (Hu and Bentler, 1999), supporting the adequacy of the three-factor measurement model.

The hypothesized structural model was estimated using subscale parcels as indicators. As shown in Table 7, EI significantly predicted WTC (*β* = 0.415, *p* < 0.001), indicating that learners with higher emotional intelligence were more willing to communicate in English. However, the direct effect of EI on FLLB was nonsignificant (*β* = −0.001, *p* = 0.980), suggesting that EI did not directly reduce boredom when controlling for WTC. In contrast, WTC significantly and negatively predicted FLLB (*β* = −0.153, *p* < 0.001), supporting the hypothesis that greater willingness to communicate was associated with lower boredom.

**Table 7:** Structural model (subscale parcels): standardized paths.

Outcome	Predictor	*β*	SE	*z*	*p*
FLLB	EI	−0.001	0.036	−0.025	0.980
FLLB	WTC	−0.153	0.036	−4.265	<0.001
Performance	WTC	0.242	0.031	7.860	<0.001
Performance	EI	0.150	0.030	4.950	<0.001
Performance	FLLB	−0.343	0.025	−13.558	<0.001
WTC	EI	0.415	0.028	14.573	<0.001

Standardized regression coefficients (β), standard errors (SE), *z*-values, and two-tailed *p*-values are reported. EI = Emotional Intelligence; WTC = Willingness to Communicate; FLLB = Foreign Language Learning Boredom.

**Figure 3:**
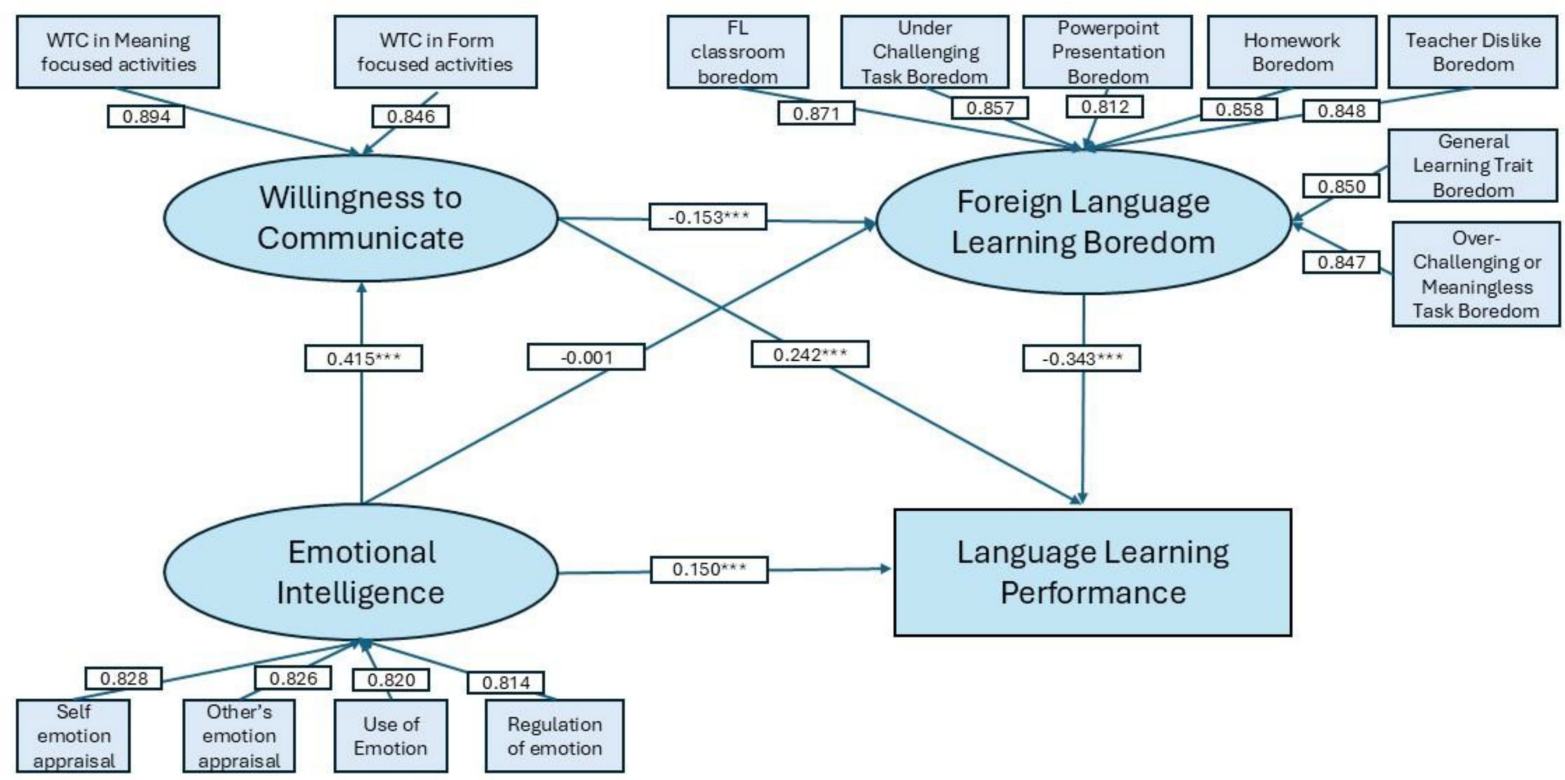
Results of the measurement and structural model.

Regarding performance outcomes, EI (*β* = 0.150, *p* < 0.001) and WTC (*β* = 0.242, *p* < 0.001) both exerted positive direct effects, whereas FLLB had a strong negative direct effect on performance (*β* = −0.343, *p* < 0.001). These results indicate that emotionally intelligent learners who were more willing to communicate and less bored tended to achieve better language learning performance. Results from both Tables 6, 7 are shown in Figure 3.

Indirect effects were examined using bootstrap resampling (Table 8). EI had a significant indirect effect on performance via WTC (ind_EI_WTC_Perf: Std. Est = 0.100, unstandardized estimate = 1.513, 95% CI [1.109, 1.984]) and a significant chain mediation through WTC and FLLB (ind_EI_WTC_FLLB_Perf: Std. Est = 0.022, 95% CI [0.179, 0.514]). The indirect path from EI to performance via FLLB alone was nonsignificant. The total indirect effect of EI on performance was significant (Std. Est = 0.122, 95% CI [1.315, 2.418]), and when combined with its direct effect, the total effect was positive and significant (Std. Est = 0.272, 95% CI [3.215, 5.000]).

**Table 8:** Structural model: indirect effects with bootstrap CIs.

Effect	Estimate	SE	*z*	*p*	Std. Est	95% CI (lower)	95% CI (upper)
ind_EI_WTC_Perf	1.513	0.227	6.675	<0.001	0.100	1.109	1.984
ind_EI_FLLB_Perf	0.005	0.184	0.026	0.980	0.000	−0.353	0.383
ind_EI_WTC_FLLB_Perf	0.328	0.085	3.870	<0.001	0.022	0.179	0.514
total_indirect	1.846	0.284	6.508	<0.001	0.122	1.315	2.418
total_effect	4.109	0.457	8.988	<0.001	0.272	3.215	5.000

Indirect effects are reported with bootstrap (B = 5,000, BCa) confidence intervals; Std. Est shows standardized effects. EI = Emotional Intelligence; WTC = Willingness to Communicate; FLLB = Foreign Language Learning Boredom. *Cis* are for Estimate.

Explained variance values are reported in Table 9. The model accounted for 17.2% of the variance in WTC and 2.3% in FLLB. Importantly, the model explained 26.1% of the variance in performance, indicating that EI, WTC, and FLLB together substantially contributed to students’ learning outcomes. High proportions of variance were also explained for the subscales, with R^2^ ranging from 0.660 to 0.799, reflecting the strong measurement quality of the parcels.

**Table 9:** Structural model: R-squared (endogenous variables).

Variable	*R^2^*
Performance	0.261
WTC	0.172
FLLB	0.023

*R*^2^ indicates the proportion of variance explained in each endogenous variable.

Finally, the global fit indices indicated that the structural model provided an excellent fit to the data: χ^2^(72) = 104.80, *p* = 0.007, CFI = 0.997, TLI = 0.996, RMSEA = 0.020 (90% CI [0.011, 0.028]), and SRMR = 0.012. All indices exceeded conventional cutoff criteria —CFI ≥ 0.95, TLI ≥ 0.95, RMSEA ≤ 0.06 with the 90% CI upper bound ≤ 0.08, and SRMR ≤ 0.08 (Hu and Bentler, 1999).

## Discussion

This study examined the relationships among emotional intelligence (EI), willingness to communicate (WTC), foreign language learning boredom (FLLB), and language learning performance in the Chinese EFL context. Using structural equation modeling with subscale parcels, the results demonstrated that EI had a significant direct effect on performance as well as indirect effects through WTC and through the sequential path WTC → FLLB. WTC positively predicted performance and negatively predicted FLLB, while FLLB strongly and negatively predicted performance. However, the direct path from EI to FLLB was nonsignificant. Overall, the model explained 26.1% of the variance in performance, underscoring the importance of both emotional and communicative variables in second language learning outcomes.

H1 predicted that EI would be positively associated with performance. This hypothesis was supported: controlling for WTC and FLLB, EI showed a significant positive direct effect on language learning performance. Within Control–Value Theory (Pekrun, 2006), this direct path suggests that learners with higher EI sustain more favorable control (perceived competence/agency) and value (importance/interest) appraisals toward language tasks, thereby fostering positive achievement emotions and more effective self-regulation that translate directly into higher test performance—even after accounting for communicative engagement (WTC) and boredom (FLLB) (Li, 2020; Li C., 2025). The finding aligns with reports that EI contributes to L2 achievement via enhanced emotion regulation and self-regulated learning (e.g., Taherkhani and Moradi, 2022; Li and Pan, 2025). The modest coefficient (*β* ≈ 0.15) is consistent with CVT’s view of EI as a distal antecedent whose benefits partly operate through appraisal-driven processes; nevertheless, the residual direct gain indicates EI confers performance advantages not fully captured by WTC or FLLB in this model (e.g., broader regulatory skills, goal maintenance, adaptive attribution).

H2 proposed that EI would be positively associated with WTC. This was confirmed: EI had a strong positive effect on WTC. The model explained 17.2% of the variance in WTC. Students who accurately recognize and label their own feelings can pre-empt maladaptive reactions (e.g., shyness, momentary withdrawal) and lean into idea exchange, storytelling, and opinion-giving that dominate meaning-focused talk (Birjandi and Tabataba'ian, 2012; Gao et al., 2025). Meanwhile, accurately reading peers’ and teachers’ emotional cues lowers perceived social risk in open discussion (Piccerillo et al., 2025). By sensing receptivity, enthusiasm, or impatience, learners can time their entries, align tone, and build on peers’ ideas—behaviors that raise willingness to join meaning-focused exchanges (Dewaele and Pavelescu, 2021). The positive association aligns with studies showing that EI supports communicative engagement and WTC by enabling learners to manage emotions and sustain confidence (Li and Pan, 2025). Our standardized coefficient (*β* = 0.415) is on the upper end of effects reported in some earlier work, which may reflect the EFL context (large classes, exam pressure), in which learners with higher EI may derive more perceived control and value from communicative opportunities (Li and Pan, 2025; Taherkhani and Moradi, 2022).

H3 predicted that WTC would be negatively associated with FLLB. In the structural model, this hypothesis was supported. WTC negatively predicted FLLB. The proportion of variance explained in FLLB was modest, reflecting that boredom is multiply determined in classroom settings. The direct path from EI to FLLB was non-significant, indicating that WTC—not EI per se—accounts for the statistically reliable reduction in boredom in our model. The small R^2^ for FLLB is also consistent with prior observations that boredom is multi-determined—shaped not only by learners’ engagement tendencies but also by task design (over−/under-challenge), teacher behaviors, monotony, and trait-like boredom proneness (Li, 2021).

H4 posited that FLLB would be negatively related to performance. This was supported. FLLB showed a robust negative effect on performance. Boredom typically reflects low perceived value (e.g., tasks seen as uninteresting/meaningless) and/or mismatched control (e.g., under- or over-challenge) (Li, 2021). Such appraisals undermine sustained attention, reduce cognitive investment, and dampen self-regulatory effort—mechanisms that directly compromise learning efficiency and test performance (Li C. et al., 2023). The sizable negative path (*β* = −0.343) aligns with CVT’s prediction that low control–low value appraisals precipitate boredom, which in turn suppresses outcomes. Notably, the effect remains strong even after accounting for EI and WTC, indicating that boredom exerts a unique, proximal drag on achievement beyond learners’ emotional competencies and their willingness to speak.

H5 is also supported. WTC positively predicted performance. Consistent with the model structure, WTC also contributed indirectly to better outcomes through lower boredom: WTC → FLLB was negative, and FLLB → performance was negative, implying a small yet favorable WTC → FLLB → performance pathway in addition to the direct effect. According to Control–Value Theory, learners’ willingness to communicate reflects how much control they feel over language use and how much value they attach to interaction. Students who believe they can express themselves effectively and who view classroom communication as meaningful are naturally more inclined to speak. Such confidence and perceived relevance encourage them to engage actively—asking questions, practicing more often, and responding to feedback—which ultimately enhances their language performance. Moreover, active participation helps maintain a sense of control and purpose during lessons, preventing the decline in interest and attention that leads to boredom, a typical low-control, low-value emotion. In this way, WTC not only promotes engagement but also indirectly protects learners from boredom and supports achievement. The present result is consonant with the WTC literature that links readiness to speak with greater L2 use and achievement (Sun et al., 2024).

H7 proposed that EI would indirectly affect performance through the sequential mediation of WTC and FLLB. This was supported. Bootstrap tests confirmed a significant chain indirect effect EI → WTC → FLLB → Performance. According to Control–Value Theory (Pekrun, 2006), emotions such as boredom arise when students treat tasks as having low value or being under/over challenging (low control). Learners with high EI are more capable of generating favorable appraisals, regulating negative emotions, sustaining optimism, and perceiving value in communication, which directly translates into higher WTC (Gao et al., 2025). Greater WTC then functions as a proximal behavioral buffer against boredom: by actively engaging in communicative tasks, learners reinforce their sense of control and discover meaning in classroom activities, thus counteracting the disengagement that underlies boredom.

Although the chain mediation effect (EI → WTC → FLLB → Performance) was statistically significant, its magnitude was smaller than the more direct EI → WTC → Performance pathway. This difference is theoretically expected. The chain involves two successive mediators—WTC and FLLB—each contributing incremental variance, so the combined influence naturally attenuates across links. Moreover, boredom is a multifaceted, context-dependent emotion shaped by numerous situational factors (e.g., task monotony, instructional style, or individual boredom proneness; Li et al., 2021). As a result, EI’s indirect influence on performance through both WTC and FLLB is necessarily smaller but still meaningful, reflecting how emotional competence enhances engagement, which then helps regulate classroom emotions, ultimately improving learning outcomes.

This pathway also helps explain why the direct EI → FLLB effect (H6) was not significant in the model. Once WTC is included, the influence of EI on boredom is largely indirectly operating through engagement behaviors rather than direct emotional suppression. This pattern echoes recent findings that boredom is multi-determined, shaped not only by individual traits but also by task design and interactional dynamics (Li, 2021). The small but significant chain effect suggests that EI’s benefits extend beyond engagement itself, reaching into the emotional climate of the classroom: students who are emotionally intelligent are not just more willing to speak, but their communicative engagement helps mitigate boredom, which in turn supports achievement. Prior work on EI and WTC (Birjandi and Tabataba'ian, 2012; Taherkhani and Moradi, 2022; Zou and Park, 2024) has emphasized the positive role of emotional competencies in sustaining communication, while research on boredom (Li et al., 2021) has consistently documented its negative effect on learning outcomes. By integrating these strands, the present results extend the literature by providing evidence for a sequential, CVT-consistent mechanism: EI → WTC → FLLB → Performance.

The present findings can also be interpreted through the lens of broader SLA frameworks, particularly Dörnyei’s L2 Motivational Self System and Complex Dynamic Systems Theory (CDST). Within Dörnyei’s framework, learners’ willingness to communicate can be seen as a behavioral manifestation of their ideal L2 self and ought-to self: students who can imagine themselves as successful L2 users are more likely to seek out and enter communicative opportunities, even in exam-oriented contexts. Our results suggest that emotional intelligence functions as a personal resource that supports this motivational system, enabling learners to regulate anxiety, sustain optimism, and maintain a sense of agency that makes the ideal L2 self more attainable in practice. In turn, higher WTC and lower boredom can be understood as proximal outcomes of a well-supported motivational self, which then facilitate better performance. From a Complex Dynamic Systems perspective, the relationships among EI, WTC, FLLB, and performance are unlikely to be strictly linear or unidirectional. Rather than viewing EI, motivation, WTC, and boredom as static traits, CDST invites us to conceptualize them as interacting subsystems that fluctuate over time and across tasks. Our structural model captures one theoretically plausible pathway—EI → WTC → FLLB → performance—but CDST implies that, in real classrooms, spikes in boredom may also dampen WTC, and repeated successes in communication may gradually reshape learners’ emotional patterns and self-concepts. Thus, the present study offers a snapshot of one dominant attractor state in this complex system, showing how emotional resources and communicative readiness can coalesce to support achievement.

### Theoretical and pedagogical implications

This study extends EI theory (Wong and Law, 2002) to the Chinese EFL context by showing that EI influences not only general academic outcomes but also communicative readiness and emotional experience. The strong EI–WTC link enriches both EI and WTC theories (MacIntyre et al., 1998; Peng and Woodrow, 2010). Incorporating FLLB advances the growing body of research on negative emotions in SLA (Li, 2021; Dewaele et al., 2023). The significant sequential mediation underscores the importance of testing complex affective–communicative pathways (Li and Pan, 2025).

Most prior studies have tested pairwise relationships (e.g., EI–WTC, WTC–performance, FLLB–performance). Our results demonstrate the value of examining chain mediations to capture the complexity of affective–communicative interactions in SLA. Theoretically, this suggests that learners’ emotions and communication behaviors are interdependent processes that jointly shape outcomes, supporting calls for dynamic, process-oriented approaches to SLA affective research (MacIntyre and Gregersen, 2012a; MacIntyre and Gregersen, 2012b; Macintyre et al., 2019).

The results suggest several pedagogical implications. Firstly, enhancing students’ emotional intelligence (EI) may indirectly boost performance by increasing WTC. To this end, social–emotional learning (SEL) programs can be integrated into the curriculum, helping learners to recognize, understand, and regulate their emotions. Teachers can also engage in empathy training and reflective teaching practices, which foster emotionally supportive classroom climates and model effective emotion regulation for students. Secondly, promoting learners’ willingness to communicate requires concrete opportunities for authentic interaction. Beyond encouraging participation in class discussions, teachers can introduce peer dialogue journals, role-play tasks, and student-led discussions, which give learners more ownership of their communicative practices. Establishing extracurricular speaking clubs or language cafés may also help students develop confidence and reduce communication anxiety. Lastly, the chain mediation results suggest that interventions targeting WTC can simultaneously reduce boredom and enhance performance. For instance, structured classroom activities that gradually increase communicative demands while providing scaffolding may help learners sustain control and value appraisals, thereby minimizing boredom and fostering achievement.

### Limitations and future directions

There are some limitations in this study. Firstly, the study relied primarily on self-report questionnaires to measure emotional intelligence, willingness to communicate, and foreign language learning boredom. While the selected scales have demonstrated reliability and validity, self-reported data may be subject to social desirability bias and inaccuracies in introspection. The use of self-reported course grades presents an additional limitation. Because the study employed full anonymity, students manually entered their own final English course grades, and no external verification was possible. Furthermore, course grades may vary across institutions and include components beyond linguistic proficiency (e.g., attendance or participation). Future studies may incorporate standardized or institution-verified proficiency measures to strengthen construct validity while following the protocol of anonymity.

Secondly, the study employed a cross-sectional design, which limits the ability to draw causal inferences. Although structural equation modeling allowed us to test hypothesized relationships, longitudinal or experimental studies are necessary to confirm the temporal ordering of EI, WTC, FLLB, and performance. Future research could incorporate multi-method approaches, such as classroom observations, peer/teacher ratings, or experimental tasks, to triangulate self-reported measures of EI, WTC, and FLLB. This would provide a more comprehensive understanding of learners’ emotional and communicative profiles. Furthermore, longitudinal studies are recommended to track changes in EI, WTC, and FLLB over time and to test causal pathways. For instance, future work could investigate how interventions aimed at enhancing EI influence WTC and reduce FLLB across a semester or academic year.

Thirdly, this study used samples from pure Chinese EFL settings, the results might be intertwined with some Chinese cultural features and in this study, we used item parceling in the SEM analysis, which might also give rise to some bias. In terms of samples, future studies might also test the results from this study in students selected from non-Chinese EFL settings. In respect of methods, future studies can also compare the SEM results generated from parceled and non-parceled item indicators.

Fourthly, although the study was conceptually grounded in Control–Value Theory (CVT), we did not directly measure learners’ control or value appraisals. Our structural model was specified in a way that is consistent with CVT, but it does not constitute a direct empirical test of the full control–value process. References to CVT should be understood as theoretical interpretations of the observed relations among EI, WTC, FLLB, and performance, rather than as evidence that specific control and value appraisals were causally involved. Future research could incorporate explicit measures of perceived control and task value or broaden the outcome variables beyond test scores to include speaking performance, classroom participation, and long-term motivation. By integrating both cognitive and affective outcomes, future studies may offer richer pedagogical insights into how emotional intelligence contributes to successful language learning.

Finally, although the present study conceptualized WTC as a proximal behavioral tendency that may reduce subsequent boredom, prior SLA research also suggests that boredom and other emotions can influence learners’ willingness to communicate. Because our cross-sectional design cannot determine temporal precedence or test reciprocal relationships, the chosen sequence (WTC → FLLB) should be interpreted as one theoretically informed pathway rather than a definitive causal ordering. Future longitudinal or experience-sampling studies are needed to examine whether WTC and boredom mutually influence each other over time.

## Conclusion

This study demonstrates that EI enhances language performance both directly and indirectly by fostering WTC and reducing FLLB. WTC emerged as the critical mediator in this process, highlighting its role in bridging emotional competencies and learning outcomes. These findings deepen our theoretical understanding of affective and communicative factors in SLA and provide practical guidance for fostering emotional and communicative engagement in EFL classrooms.

## Data Availability

The raw data supporting the conclusions of this article will be made available by the authors, without undue reservation.
